# Does Playing Apart Really Bring Us Together? Investigating the Link Between Perceived Loneliness and the Use of Video Games During a Period of Social Distancing

**DOI:** 10.3389/fpsyg.2022.683842

**Published:** 2022-02-11

**Authors:** Steve Nebel, Manuel Ninaus

**Affiliations:** ^1^Chair of Psychology of Learning With Digital Media, Faculty of Humanities, Institute of Media Research, Chemnitz University of Technology, Chemnitz, Germany; ^2^Department of Clinical Psychology, Institute of Psychology, University of Innsbruck, Innsbruck, Austria; ^3^Leibniz-Institut für Wissensmedien, Tübingen, Germany; ^4^LEAD Graduate School and Research Network, University of Tübingen, Tübingen, Germany; ^5^Institute of Psychology, University of Graz, Graz, Austria

**Keywords:** gaming frequency, loneliness, gaming motives, social gaming, COVID-19, mental health

## Abstract

During the COVID-19 pandemic, several countries implemented social distancing measures to contain virus transmission. However, these vital safety measures have the potential to impair mental health or wellbeing, for instance, from increased perceived loneliness. Playing social video games may offer a way to continue to socialize while adhering to social distancing measures. To examine this issue further, the present online survey investigated social gaming during the pandemic and its association to perceived loneliness within a German-speaking sample. Results indicated a small positive correlation between general gaming frequency and perceived loneliness. Detailed analysis revealed a negative association between perceived loneliness and increased social forms of video gaming. Specifically, gamers with a higher social motive for gaming perceived less loneliness, but gamers with a dominant escape motive demonstrated a positive link to perceived loneliness. The use of social gaming in times of social distancing seems to play a small but significant factor in perceived loneliness compared to other demographical data. The findings are discussed with respect to methodological limitations, effect sizes, and sample characteristics. The results enrich the current knowledge on video gaming and its link to social wellbeing and provide a more nuanced picture than simplistic investigations of screen time.

## Introduction

In 2020 the world was hit by the coronavirus pandemic, which disrupted our everyday lives. To reduce the transmission of COVID-19, numerous countries imposed severe measures, such as “lockdowns” that closed commercial, cultural, and social venues and stay-at-home mandates, to facilitate social distancing (e.g., Bundesministerium für Gesundheit, 2020). Video game industry partners launched the initiative #PlayApartTogether (e.g., Johnston et al., 2020), which encouraged the use of video games for socializing to promote social distancing initiatives and to help counteract the potential negative side effects of social distancing, such as anxiety, depression, and loneliness, that people may experience during the current pandemic from the decrease in socialization (APA, 2020; WHO, 2020). Indeed, research has suggested that playing video games is significantly associated with mental health and wellbeing (e.g., Jones et al., 2014; Johannes et al., 2021). Further studies indicated that playing video games causally improves players’ moods and promotes relaxation (e.g., Russoniello et al., 2009; Bowman and Tamborini, 2015; Greitemeyer et al., 2019; Pine et al., 2020) and may reduce loneliness (Colder Carras et al., 2017).

The investigation of social gaming based on individual social factors is not a new area of scholarly interest (e.g., Kowert et al., 2014; Kowert and Oldmeadow, 2015; Kaye et al., 2017; for a recent and comprehensive overview see Kaye, 2021), but the subject has gained considerable importance during the pandemic, as social distancing measures may exacerbate loneliness and social isolation. Both aspects have been linked to poor health outcomes and may increase the risk of depression and anxiety (Santini et al., 2020). The use of video games may be one way to foster social connectedness in times of global quarantines (Marston and Kowert, 2020). Video games played online can facilitate social interactions that are crucial for our social wellbeing (e.g., Depping and Mandryk, 2017; Depping et al., 2018), and if players engage in gaming freely, authentically, and in balance with other activities or goals in their lives, it may be capable of reducing loneliness (Mandryk et al., 2020). In this vein, other researchers have suggested that social games may enable players to feel more connected during times of isolation and difficult life experiences in general (Iacovides and Mekler, 2019). Accordingly, behavioral science advisors have recommended that free access to gaming be provided to ease the negative impacts of self-isolation (Independent Scientific Pandemic Insights Group on Behaviours, 2020a,b). However, researchers acknowledge that problems can arise if games are used only as a tactic to avoid critical problems or as an escape mechanism (for a more comprehensive discussion see Király et al., 2017); nevertheless, they may help players cope with challenging situations (Iacovides and Mekler, 2019). Therefore, the way individuals use games and their dominant motives for gaming may be determining factors that can be used to identify beneficial or adverse effects of gaming (e.g., Király et al., 2017).

Building upon prior research on social gaming and loneliness, we aimed at investigating whether similar associations can be found during a time of mandatory social isolation—as happening during particular phases of the pandemic. Further, in contrast to broad descriptions of “screen time” or gaming, the current study aimed at providing a differential perspective by not only investigating different modes of gaming (i.e., single-player vs. multiplayer) but also different motives for gaming. In response to recent calls for transparency in the social sciences (e.g., Vazire, 2017) and gaming research (Vornhagen et al., 2020), we strived for a transparent workflow and made our data and analysis code openly (Nebel and Ninaus, 2020) available so that others can easily build upon our research.

## Materials and Methods

The present study belongs to a larger project investigating the effects of social distancing on gamers during the pandemic. The survey was designed to gather a convenience sample including as many active video gamers as possible. Such a sample, however, may not make it possible to reach valid conclusions, as regional and chronological differences in the governmental handling of the pandemic have been quite substantial. To avoid this issue, a more homogenous sample relative to the social distancing measures imposed was aimed for, and both the data collection period and target sample population were narrowed. More specifically, data collection was limited to German-speaking participants and to 18 days starting in mid-April 2020. This data acquisition period was selected because it allowed for the collection of data from a period when comparable pandemic measures were taken across the targeted German-speaking countries. At that time, Germany and Austria were undergoing a partial lockdown (e.g., Bundesministerium für Gesundheit, 2020), while Switzerland was still under a lighter lockdown. That is, data were acquired during a time when most participants were not allowed to physically meet other people outside of their household or could only do so under very strict conditions.

### Participants and Procedure

Adult participants were recruited from social networks (e.g., Twitter), gaming forums,^1^ and as a result of general media coverage, a popular German gaming magazine highlighted the study in their news section (e.g., Bathge, 2020). Upon clicking the survey link, participants received information on the goals, content, potential risks, and privacy regulations of the survey and were asked for consent regarding data collection and publication, anonymization, study cancelation, and voluntariness. In addition, they had to confirm that they were at least 18 years old. At the end of the survey, participants were thanked and provided with web links to contact points and offer help with mental problems that can arise from the lack of social interaction.^2^^,^^3^ Out of 1,150 participants who started the survey, 783 participants completed it in full (68% response rate), but 41 participants were excluded from further analyses, as they failed to respond correctly to our three instructed response items (e.g., “Please respond with a three for this item”; cf. Meade and Craig, 2012). One additional participant had to be excluded because he/she had never played a video game, and having played video games at least once in the prior month was a requirement for participating. The final sample analyzed comprised *N* = 741 respondents. The study was approved by the ethics commission at the Leibniz-Institut für Wissensmedien, Tübingen, Germany (LEK 2020/017).

### Measures

#### Sociodemographic and Pandemic-Related Variables

In addition to the collection of basic sociodemographic data, including age, gender, marital status, and current employment status, we assessed several variables to better understand the current situation of the participants during the pandemic. To allow for future sample comparisons across different COVID-19 related studies, we aligned our assessed variables with the COVIDiSTRESS global survey (Lieberoth et al., 2020). In particular, we collected data on (i) whether participants currently live in their home country, (ii) the country they currently live in, (iii) whether they had close relatives or friends within a high-risk group regarding COVID-19, (iv) whether their current lifestyle was the same as the lifestyle they followed before the pandemic, (v) whether they live alone or with other adults and/or children, and (vi) how much time they currently have for leisure activities. This alignment should support future comparisons. However, due to the alignment, some special regional characteristics could not be included in the questionnaire, such as the German government supporting Kurzarbeit, which is time-limited part-time employment. This classification refers to employees who are technically full-time employees but are temporarily working part-time during the pandemic. Since such peculiarities were not included in the COVIDiSTRESS (Lieberoth et al., 2020) questionnaire, they were not included in this survey, either.

#### Gaming Frequency

Participants’ gaming frequency at the time they completed the survey was assessed on a 7-point ordinal scale (1 = never; 2 = several times a month; 3 = several times a week; 4 = daily; 5 = more than 1 h daily; 6 = more than 3 h daily; and 7 = more than 6 h daily). As a consequence of this classification, the resulting scale is not linear and was suitable to avoid ceiling effects when only using “daily” as the maximum category.

#### Change in-Game Behavior

Participants were asked to respond to the following question regarding changes in gaming behavior due to the pandemic: “Compared to the time before the COVID-19 pandemic, how much time do you currently spend on …” (1) video games in general (*M* = 3.73, *SD* = 0.88), (2) single-player video games (*M* = 3.35, *SD* = 0.91), (3) cooperative video games (*M* = 3.37, *SD* = 0.86), (4) competitive video games (*M* = 3.18, *SD* = 0.74), and (5) online games to stay in contact with friends (*M* = 3.47, *SD* = 0.89). Participants responded to these questions on a 5-point Likert scale (ranging from “1 = much less than before” to “5 = much more than before”). Naturally, this variable cannot represent absolute playtime, but relative self-reported changes in playtime, i.e., someone playing excessively before the pandemic, but reducing playtime during this time might report a low value. In contrast, a player rarely plays at all, but increasing playtime during this time might submit a high value.

#### Motives for Gaming

Motives for gaming were measured using subscales from the motives for online gaming questionnaire (Demetrovics et al., 2011), which were translated to German. For the current study, we used the following subscales: *social* (e.g., “. because I can meet many different people,” Cronbach’s α = 0.77), *escape* (e.g., “. because gaming helps me to forget about daily hassles,” α = 0.85), *competition* (e.g., “. because I enjoy competing with others,” α = 0.83), *coping* (e.g., “. because it helps me get rid of stress,” α = 0.74), *skill development* (e.g., “. because it improves my skills,” α = 0.91), and *fantasy* (e.g., “. because I can be in another world,” α = 0.81). Each subscale was assessed using four items, which were rated on a 5-point Likert scale (ranging from “1 = almost never/never” to “5 = almost always/always”).

#### Loneliness

To assess participants’ experienced loneliness we used a German translation of the UCLA Loneliness Scale (Russell, 1996), which includes 20 items, to which participants respond using a 4-point Likert scale (ranging from “1 = never” to “4 = always”; α = 0.91). Examples of questions from the survey include “How often do you feel that you lack companionship?” and “How often do you feel that there are people you can talk to?” (reverse coded).

#### Analysis

The analysis was conducted using SPSS Statistics (IBM, 2020), R (R Core Team, 2020), and the R packages psych (Revelle, 2020), ggplot2 (Wickham, 2016), and GGally (Schloerke et al., 2020). The dataset and analysis code are publicly available (Nebel and Ninaus, 2020).

## Results

### Sample Description

To provide a sufficient understanding of the sample and to enable the results to be put into perspective, the sample is described in detail (see Table 1). The mean age was 31.75 (*SD* = 9.04), ranging from 18 to 75. The majority of the sample (95.7%) had kept their employment status during the pandemic until the time of the survey. Likewise, most of the sample (95.4%) lived within their home country. Over half of the participants (52.1%) reported that at least one of their close relatives or friends could be considered in the high-risk group regarding COVID-19, while 38.2% reported that this was not the case, and the remaining 9.7% indicated they were unsure. Only 9.9% of the sample reported that they were living their usual lifestyles, while 68.3% reported that they had had to make small changes in their lifestyles, 21.7% reported a status of isolation, and one participant reported that he/she was living in isolation in a medical or similar institution. After aggregating the loneliness scales, the participants showed a low to medium sum score of 40.18 (*SD* = 10.49). This corresponds to the reported values of students (*M* = 40.08, *SD* = 9.50) and nurses (*M* = 40.14, *SD* = 9.52) in the original publication of the Scale (cf. Russell, 1996).

**TABLE 1:** Sample characteristics.

Employment status	Pupil	Student	Full-time	Half-time	Self-employed	Un-employed	Retiree
	3.0%	18.5%	61.3%	6.2%	4.3%	4.9%	1.9%
Playing video games…	Few times a month	Few times a week	Daily	More than 1 h daily	More than 3 h daily	More than 6 h daily	
	4.0%	21.6%	12.3%	23.9%	29.7%	8.5%	
Available time for leisure activities	None	Few minutes per day	1 h per day	Several hours per day	The whole day		
	0.2%	0.5%	8.4%	78.1%	12.7%		
Location	Germany	Austria	Switzerland	Luxembourg	Other		
	87.7%	7.2%	2.0%	1.1%	2%		
Living with…	No adults	One adult	Two adults	Three adults	More than three adults		
	34.5%	36.4%	14.6%	8.9%	5.6%		
	No children	One child	Two children	Three children			
	87.9%	7.6%	3.8%	0.8%			
Social status	Single	Married/relation-ship	Divorced/widowed	NA			
	51.1%	45.2%	1.9%	1.8%			
Gender	Male	Female	Non-binary	NA			
	86.6%	11.9%	0.5%	0.9%			

### Gaming Frequency

A correlation analysis was used to evaluate the association between general gaming frequency (*M* = 4.79, *SD* = 1.40) and perceived loneliness. We identified a very small but significant positive correlation between gaming frequency and loneliness (*r*_*s*_ = 0.08, 95% CI [0.01, 0.15], *p* = 0.025).

### Purpose of the Game

**FIGURE 1:**
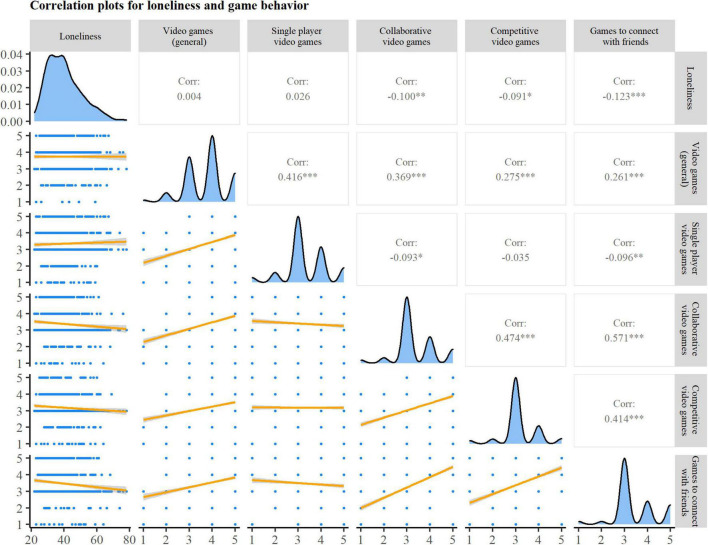
Correlation between loneliness and change in game behavior due to the COVID-19 pandemic. Upper triangle: Correlation coefficients. Significant correlations are marked with asterisks (*p < 0.05; **p < 0.01; ***p < 0.001). Diagonal: Density plot of individual variables. Lower triangle: Scatterplots for each correlation; gray shaded area indicates 95% confidence region for the correlation.

To better understand the association between gaming frequency and loneliness, we ran a correlation analysis between the individual gaming behavior changes and loneliness. The results demonstrated a small negative link between increased social forms of gaming during the pandemic and loneliness, ranging from (*r*_*s*_ = –0.09) to (*r*_*s*_ = –0.12; see Figure 1.) In contrast, no significant correlations were observed between increased general play frequency or increased single-player games playtime and loneliness.

### Player Motive

**FIGURE 2:**
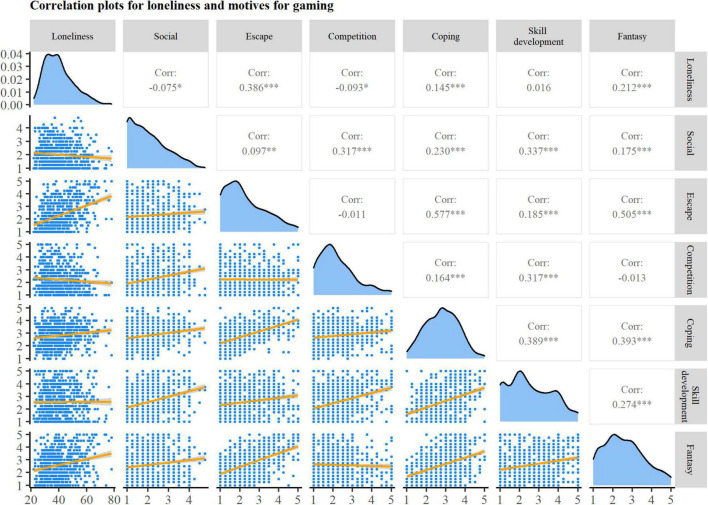
Correlation plot between loneliness and motives for gaming. Upper triangle: Correlation coefficients. Significant correlations are marked with asterisks (*p < 0.05; **p < 0.01; ***p < 0.001). Diagonal: Density plot of individual variables. Lower triangle: Scatterplots for each correlation; gray shaded area indicates 95% confidence region for the correlation.

**FIGURE 3:**
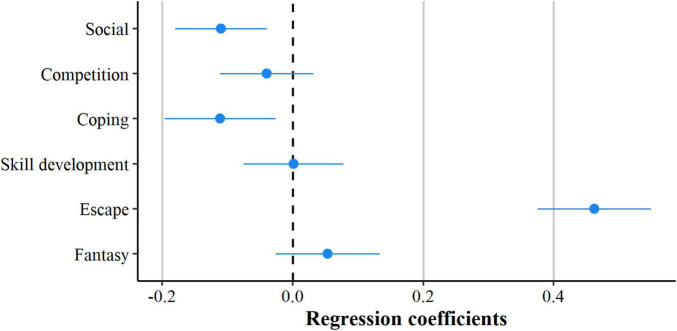
Standardized beta coefficients from the multiple linear regression model predicting loneliness. Error bars indicate 95% CIs.

To evaluate how general motives for gaming are related to loneliness, correlation analyses were run. These analyses shed light on the associations between different frequencies of and motives for gaming (see Figure 2). As a second step, a forced-entry multiple regression analysis was run to predict participants’ loneliness based on their general motives for gaming. Standardized parameters were obtained by fitting the model on a *z*-standardized version of the dataset. Effect sizes were labeled following Cohen’s (1988) recommendations. The model explained a significant and moderate proportion of variance [*R*^2^ = 0.20, *F*(6, 734) = 31.32, *p* < 0.001, *adj. R*^2^ = 0.20]. Results of the regression analysis (see Figure 3) indicated that the effect of the *social* motive was negative and could be considered very small and significant (*std.*β = –0.11, *SE* = 0.04, 95% CI [–0.18, –0.04], *p* = 0.002). The effect of the motive *escape* was positive and can be considered small and significant (*std.*β = 0.46, *SE* = 0.04, 95% CI [0.38, 0.55], *p* < 0.001). The effect of the *coping* motive was negative and can be considered very small and significant (*std.*β = –0.11, *SE* = 0.04, 95% CI [–0.20, –0.03], *p* = 0.01). The very small and not significant effects of the motives *competition* (*std.*β = –0.04, *SE* = 0.04, 95% CI [–0.11, 0.03], *p* = 0.276), *skill development* (*std.*β = 0.00, *SE* = 0.04, 95% CI [–0.08, 0.08], *p* = 0.973), and *fantasy* (*std.*β = 0.05, *SE* = 0.04, 95% CI [–0.03, 0.13], *p* = 0.190) did not account for a unique part of the variance of the loneliness that participants experienced.

### Putting Effect Sizes Into Perspective

To put results into perspective, we present exemplary effects between descriptive data and loneliness. For instance, a similar effect in comparison to the small effect characterizing the positive correlation between game frequency and loneliness was also identified between time for leisure activities and loneliness (*r*_*s*_ = 0.10, 95% BCa CI [0.03, 0.18], *p* = 0.006). Negative effects on the amount of perceived loneliness, such as the negative connection between the increased social game use and loneliness, were also identified between the number of children below the age of 12 in the household and loneliness (*r*_*s*_ = –0.08, 95% BCa CI [–0.16, –0.01], *p* = 0.03). The largest observed connection between the *escape* motive and loneliness was close to a medium effect size. However, even this effect appeared small, compared to some effect sizes within the descriptive data of the sample. More specifically, the difference of being single or in a relationship on loneliness (*U* = 41684.00, *Z* = –7.91, *p* < 0.001, *d* = 0.62, 95% BCa CI [0.47, 0.77]) exceeds such medium-sized effects.

## Discussion

The survey data uncovered details on the link between reported loneliness, change in gaming behavior and gaming frequency during a time of social distancing. Positive connections between loneliness and gaming frequency only appeared to be unambiguous on a superficial level. Upon closer investigation, both the change in usage of a specific type of game and the motive behind why the game is played revealed a much more nuanced picture. In contrast to overall gaming frequency or increased gaming behavior during the pandemic (which revealed no significant connection to loneliness), increased social forms of gaming during the pandemic were linked to lowered perceptions of loneliness. The significant results within the relative self-reported measures of change of gaming behavior could indicate that a shift toward specific games could be beneficial for wellbeing, independently from the overall gaming frequency. This could lead to useful interventions for all types of gamers. These insights should be further investigated in combination with research on motivational factors. For instance, experienced autonomy was revealed as a positive predictor of wellbeing, and extrinsic motivations as a negative predictor (Johannes et al., 2021). It remains to be seen if positive effects of changes toward social games could be enhanced if those games would focus on autonomous actions as well. At the same time, the positive effect might be dampened if an external factor initiated this change. A more fine-grained analysis of social aspects is needed, as different aspects of social games might induce individual effects (Kaye, 2021).

Different motives for gaming seem to be correlated with increased (e.g., *escape*) or decreased (e.g., *social*) loneliness. These findings are in line with previous studies that indicated a positive association between escape motives and the problematic use of gaming (e.g., Király et al., 2017). Importantly though, it has been shown that escape motives are only negatively associated with wellbeing in case players also reported psychological difficulties such as high stress and low self-esteem (Kardefelt-Winther, 2014). Gamers with good mental health may also play for escape motives but without experiencing potential negative consequences (for a comprehensive discussion see Király et al., 2017). The factor *coping* represents an especially interesting case, both from a theoretical standpoint (as the measured items are very similar to the topic of mood management within problematic media use research) as well as from a statistical perspective. If addressed individually, *coping* seems to be positively related to loneliness; if investigated in a model including all gaming related motives, it demonstrates a negative relationship. This indicates sub-facets or complex connections to other motives not yet sufficiently mirrored within the measures. This is in line with qualitative research, revealing manifold variations of coping strategies using games, such as socializing, respite, avoidance, distancing, encouragement for other activities or meaning-making (Iacovides and Mekler, 2019).

Overall, this analysis indicated beneficial game motives with respect to loneliness (e.g., *social*). Current results, acquired during a time of mandatory social isolation, are in line with the overall sentiment of previous studies suggesting that social gaming can facilitate social interaction and might combat loneliness by, for instance, connecting us to strangers or support maintaining existing relationships (e.g., Dabbish, 2008; Depping et al., 2018; Mandryk et al., 2020). Also, it revealed a more nuanced perspective on motives that are considered potentially harmful (e.g., *coping*) within research on problematic media usage (e.g., Lemmens et al., 2009). However, future studies should verify whether the results are specific to times of social-distancing. In addition, methods used for this investigation were based on linear connections, although non-linear links cannot be ruled out. Thus, broad-brushed conclusions should be avoided. The observed effects were small but in line with similar studies. For instance, an effect of β = 0.1 has been observed between game time and wellbeing (Johannes et al., 2021). As demonstrated within the “Putting Effect Sizes into Perspective” chapter, similar links can be identified with other variables as well. This indicates a relevant but not dominant connection between video gaming and perceived loneliness, especially compared to more important links, such as loneliness and relationship status. This should be kept in mind when deriving real-world implications. For instance, social games could be one element of a useful support strategy during such a crisis, whereas relying on them alone may be insufficient. Moreover, social gaming is a complex phenomenon and not all aspects of it could be studied in the current study (for a recent overview and framework see Kaye, 2021).

The description of the sample within this research report should be kept in mind when discussing the observed effects. The majority of the participants could spend at least a few hours a day on leisure activities, which is not the case for everybody, during a pandemic or in general. It seems reasonable to assume that the effects may be different in a sample including such subjects. Even if identical, the practical application might be a difficult endeavor. For instance, parents occupied with childcare suffered extra workload due to closed schools during the pandemic. As a result, they may not be able to support their mental health with time-consuming video games and might not have had the time to partake in the current study. Furthermore, the game choice is also influenced by other aspects undoubtfully influenced by the pandemic, such as boredom or stress (Bowman and Tamborini, 2015). Also, this survey cannot provide long-term insights. It is not clear how the observed connections evolve or can be evaluated against other effects. For instance, it may be advisable to accept minor negative effects to prevent stronger long-term impairments.

The convenience sample is not representative of the population within the targeted countries. This gains importance during a pandemic. Although it was essential to collect data during the potentially isolating lockdown period to gather information on loneliness specifically under these circumstances, it is natural that a large part of the population did not have the capacity to participate in this research and is, therefore, not reflected within the data. Additionally, some measures are traditionally problematic. For instance, in a recent study, participants overestimated their game time by 2 h (Johannes et al., 2021). Finally, it should be clearly stated that most analyses are based on correlations and cross-sectional data. Thus, we cannot infer cause and effect but only report similarities or differences appearing to be significant from a statistical perspective. The underlying mechanisms are most certainly much more complex, and caused, mediated, or moderated by variables not reflected in our survey. It remains to be seen, if results can be replicated or expanded in different (technological) cultures, under different social restrictions, or using samples with different demographic characteristics. However, the size of the collected sample, the clear pattern within the observed effects, and the similarity to other previous research should be sufficient to be optimistic about the use of social gaming during challenging periods and to motivate further desperately needed experimental investigations. Especially, as the effects emerged, even though over 90% of the sample could not live their life as usual. In conclusion, this survey provides more nuanced insights that may be useful as an additional argument against one-sided fearmongering pertaining to the potentially harmful effects of video games. Further, it emphasizes the need for a differential view on various forms of and motives for gaming. Specifically, observed results emphasize the need for a more careful analysis and understanding of video gaming behavior in contrast to potentially superficial screen time or frequency investigations.

## Data Availability Statement

The datasets presented in this study can be found in online repositories. The names of the repository/repositories and accession number(s) can be found below: https://osf.io/jdxyr.

## Ethics Statement

Ethical review and approval was not required for the study on human participants in accordance with the local legislation and institutional requirements. The patients/participants provided their written informed consent to participate in this study.

## Author Contributions

SN: conceptualization, methodology, formal analysis, investigation, data curation, writing—original draft, writing—review and editing, supervision. MN: conceptualization, methodology, validation, formal analysis, investigation, resources, data curation, writing—original draft, writing—review and editing, visualization, supervision. Both authors approved the submitted version.

## Conflict of Interest

The authors declare that the research was conducted in the absence of any commercial or financial relationships that could be construed as a potential conflict of interest.

## Publisher’s Note

All claims expressed in this article are solely those of the authors and do not necessarily represent those of their affiliated organizations, or those of the publisher, the editors and the reviewers. Any product that may be evaluated in this article, or claim that may be made by its manufacturer, is not guaranteed or endorsed by the publisher.

## Footnotes

1
https://www.4players.de/

2
dgppn.de/schwerpunkte/corona-psyche.html

3
infektionsschutz.de/coronavirus/psychische-gesundheit.html
